# Effectiveness of Modified Vaccinia Ankara-Bavaria Nordic Vaccination in a Population at High Risk of Mpox: A Spanish Cohort Study

**DOI:** 10.1093/cid/ciad645

**Published:** 2023-10-21

**Authors:** Mario Fontán-Vela, Victoria Hernando, Carmen Olmedo, Ermengol Coma, Montse Martínez, David Moreno-Perez, Nicola Lorusso, María Vázquez Torres, José Francisco Barbas del Buey, Javier Roig-Sena, Eliseo Pastor, Antònia Galmés Truyols, Francisca Artigues Serra, Rosa María Sancho Martínez, Pello Latasa Zamalloa, Olaia Pérez Martínez, Ana Vázquez Estepa, Amós José García Rojas, Ana Isabel Barreno Estévez, Alonso Sánchez-Migallón Naranjo, Jaime Jesús Pérez Martín, Pilar Peces Jiménez, Raquel Morales Romero, Jesús Castilla, Manuel García Cenoz, Marta Huerta Huerta, An Lieve Dirk Boone, María José Macías Ortiz, Virginia Álvarez Río, María Jesús Rodríguez Recio, María Merino Díaz, Belén Berradre Sáenz, María Teresa Villegas-Moreno, Aurora Limia, Asuncion Diaz, Susana Monge, Spanish MPOX Vaccine Effectiveness Study Group, José Antonio Lluch Rodrigo, Juan Juaneda, Rosa Carbo Malonda, Jacobo Mendioroz, Joan Colom Farran, Fernando González Carril, Lorea Oscoz Echevarría, María Teresa Otero Barros, Nuria Suárez Gaiche, José Angélica Gómez Martínez, Borja Alcázar Bastante, Rocío Sánchez Santos, Lucía Fuster Sanjurjo, María del Carmen Pacheco Martínez, Nancy Coromoto Cruz, Laura García Hernández, Óscar Guillermo Pérez Martín, Marcos Alonso García, María Ángeles Gutiérrez Rodríguez, Beatriz López Centeno, Mario Margolles Martins, Eva Martínez Ochoa, José Luis Jiménez-Murillo, Miguel Mingo Gómez de Celis, Inma Jarrín, Bernardo Guzmán, Berta Suárez

**Affiliations:** National Centre of Epidemiology, Institute of Health Carlos III, Community of Madrid, Spain; Public Health and Epidemiology Research Group, School of Medicine and Health Sciences, Universidad de Alcalá, Alcalá de Henares, Community of Madrid, Spain; National Centre of Epidemiology, Institute of Health Carlos III, Community of Madrid, Spain; CIBER on Infectious Diseases, Madrid, Spain; Vaccination Programme, General Directorate of Public Health, Ministry of Health, Madrid, Spain; Primary Healthcare Information Systems, Health Institute of Catalonia, Catalonia, Spain; Preventive Medicine Service, General Sub-directorate for Health Promotion, Health Department, Secretariat of Public Health,Catalonia, Spain; Health and Consumption Department, General Directorate of Public Health and Pharmaceutical Management, Andalusia, Spain; Health and Consumption Department, General Directorate of Public Health and Pharmaceutical Management, Andalusia, Spain; Healthcare Department, General Sub-directorate of Health Prevention and Promotion, General Directorate of Public Health, Community of Madrid, Spain; General Sub-directorate of Public Health Surveillance, General Directorate of Public Health, Madrid, Community of Madrid, Spain; Department of Universal Healthcare and Public Health, Epidemiological Surveillance Service, Valencian Community, Spain; Universal Healthcare and Public Health Department, Health Promotion and Prevention Programs Service, Valencian Community, Spain; Disease Prevention Service, Health and Consumption Department, General Directorate of Public Health and Participation, Balearic Islands, Spain; Disease Prevention Service, Health and Consumption Department, General Directorate of Public Health and Participation, Balearic Islands, Spain; Epidemiology Unit, General Sub-directorate of Public Health and Addictions of Gipuzkoa, Basque Country, Spain; Epidemiology and Vaccination Service, General Directorate of Public Health, Basque Country, Spain; Epidemiology Service, Health Department, General Directorate of Public Health, Galicia, Spain; Epidemiology Service, Health Department, General Directorate of Public Health, Galicia, Spain; Prevention and Epidemiology Service, General Directorate of Public Health, Canarian Health Service, Canary Islands, Spain; Prevention and Epidemiology Service, General Directorate of Public Health, Canarian Health Service, Canary Islands, Spain; Epidemiology Service, Health Department, General Directorate of Public Health and Addictions, Murcia Region, Spain; Vaccination Progamme, Prevention and Health Protection Service, Health Department, General Directorate of Public Health and Addictions, Murcia Region, Spain; Epidemiology Service, Healthcare Department, General Directorate of Public Health, Castilla-La Mancha, Spain; Epidemiology Service, Healthcare Department, General Directorate of Public Health, Castilla-La Mancha, Spain; Instituto de Salud Pública de Navarra – IdiSNA – CIBERESP, Pamplona, Spain; Instituto de Salud Pública de Navarra – IdiSNA – CIBERESP, Pamplona, Spain; Vaccination Programme, Health Department, Epidemiological Surveillance Service, Principado de Asturias, Spain; Vaccination Programme, Health Department, Epidemiological Surveillance Service, Principado de Asturias, Spain; Vaccination Program, General Directorate of PublicHealth, Healthcare Service of Extremadura, Spain; Epidemiology Service, Healthcare Department, General Directorate of Public Health, Castilla y León, Spain; Epidemiology Service, Healthcare Department, General Directorate of Public Health, Castilla y León, Spain; Epidemiology and Healthcare Prevention Service, Health Department, General Directorate of Public Health, Consumption and Nursing, La Rioja, Spain; Epidemiology and Healthcare Prevention Service, Health Department, General Directorate of Public Health, Consumption and Nursing, La Rioja, Spain; National Centre of Epidemiology, Institute of Health Carlos III, Community of Madrid, Spain; Vaccination Programme, General Directorate of Public Health, Ministry of Health, Madrid, Spain; National Centre of Epidemiology, Institute of Health Carlos III, Community of Madrid, Spain; CIBER on Infectious Diseases, Madrid, Spain; National Centre of Epidemiology, Institute of Health Carlos III, Community of Madrid, Spain; CIBER on Infectious Diseases, Madrid, Spain

**Keywords:** mpox, monkeypox, vaccine effectiveness, MVA-BN vaccine, pre-exposure prophylaxis

## Abstract

**Background:**

With more than 7500 cases reported since April 2022, Spain has experienced the highest incidence of mpox in Europe. From 12 July onward, the modified vaccinia Ankara-Bavaria Nordic (MVA-BN) smallpox vaccine was offered as pre-exposure prophylaxis for those receiving pre-exposure prophylaxis for human immunodeficiency virus (HIV-PrEP). Our aim was to assess the effectiveness of 1 dose of MVA-BN vaccine as pre-exposure prophylaxis against mpox virus (MPXV) infection in persons on HIV-PrEP.

**Methods:**

National retrospective cohort study between 12 July and 12 December 2022. Individuals aged ≥18 years receiving HIV-PrEP as of 12 July with no previous MPXV infection or vaccination were eligible. Each day, we matched individuals receiving a first dose of vaccine and unvaccinated controls of the same age and region. We used a Kaplan–Meier estimator, calculated risk ratios (RR) and vaccine effectiveness (VE = [1 − RR]x100).

**Results:**

We included 5660 matched pairs, with a median follow-up of 62 days (interquartile range, 24–97). Mpox cumulative incidence was 5.6 per 1000 (25 cases) in unvaccinated and 3.5 per 1000 (18 cases) in vaccinated. No effect was found during days 0–6 post-vaccination (VE, −38.3; 95% confidence interval [CI], −332.7 to 46.4), but VE was 65% at ≥7 days (95% CI, 22.9 to 88.0) and 79% at ≥14 days (95% CI, 33.3 to 100.0) post-vaccination.

**Conclusions:**

One dose of MVA-BN vaccine offered protection against mpox in most-at-risk population shortly after the vaccination. Further studies need to assess the VE of a second dose and the duration of protection over time.

## Teaser

In the short-term, after pre-exposure administration of 1 dose of modified vaccinia Ankara-Bavaria Nordic vaccine in a high-risk population, the risk of mpox infection was reduced by 65% at ≥7 days and by 79% at ≥14 days after vaccination.

In early May 2022, an outbreak of mpox (formerly named monkeypox) emerged and rapidly spread worldwide, with more than 87 000 cases and 140 deaths reported by 111 countries 1 year later [1]. The majority were men who have sex with men [2–5]. Spain had the highest cumulative incidence of mpox in Europe and the third globally, with more than 7500 reported cases [6]. In the current outbreak, person-to-person transmission occurred predominantly through direct contact with skin lesions or with bodily fluids during sexual intercourse or prolonged close physical contact [2, 3, 7, 8].

Prevention and control measures for the current outbreak included information and awareness campaigns that involved civil society organizations, the closure of specific venues linked to mpox outbreaks (eg, saunas), and vaccination with modified vaccinia virus Ankara (MVA). Modified Vaccinia Ankara-Bavaria Nordic (MVA-BN; Bavarian Nordic, BN, branded as JYNNEOS or IMVANEX) is a third-generation vaccine against smallpox that contains a nonreplicative live virus [9]. At the beginning of the outbreak, MVA-BN vaccines were scarce and prioritized as post-exposure prophylaxis [10]. With increasing availability of vaccines, pre-exposure vaccination was recommended for persons at high risk of mpox, specifically, if they had a large number of sexual partners (≥10 in the last year or ≥3 in the previous 3 months), had been involved in group sex activities, or had a sexually transmitted infection (STI) diagnosed in the last month [11], regardless of previous vaccination against smallpox during childhood. The recommended schedule is 2 doses administered ≥28 days apart, either subcutaneous (0.5 mL) or intradermal (0.1 mL). In Spain, the first dose of MVA-BN as pre-exposure prophylaxis was started on 12 July 2022, and the second dose was started on 6 September 2022 [12].

Before the current outbreak, no estimates of clinical efficacy were available outside of animal models [13, 14]. Estimates of vaccine effectiveness (VE), when administered pre-exposure, have been generated by the United Kingdom [15], the United States [16–19], and Israel [20], though only the latter was a cohort study and had some methodological limitations [21]. The greatest difficulty for VE studies has been the lack of a sampling frame of the population targeted for vaccination, which is needed to identify groups with similar risk of mpox virus (MPXV) infection independently of the probability of receiving MVA-BN vaccination.

Individuals who receive human immunodeficiency virus pre-exposure prophylaxis (HIV-PrEP) are a well-identified population in Spain due to prescription of HIV-PrEP as part of hospital pharmacy services. These individuals are at high risk of MPXV infection and have been proactively targeted for pre-exposure MVA-BN vaccination [22]. Our aim was to estimate the reduction in the risk of MPXV infection associated with the administration of at least 1 dose of MVA-BN vaccine pre-exposure in persons receiving HIV-PrEP.

## METHODS

### Study Design and Setting

We constructed a retrospective cohort study by deterministic linkage of databases using any of 3 personal identifiers (national health system number, national identification number, and regional health system number). We collected data from 15 of 19 autonomous regions in Spain encompassing >95% of the population: Andalusia, Asturias, Balearic Islands, Canary Islands, Castile and León, Castilla-La Mancha, Catalonia, Valencian Community, Extremadura, Galicia, Community of Madrid, Region of Murcia, Navarre, Basque Country, and La Rioja. The regions reported individual-level data from 3 data sources: all diagnoses of MPXV infection, all MVA-BN vaccine doses, and the list of individuals receiving HIV-PrEP as of 12 July 2022. Individual identifiers were pseudoanonymized using a HASH algorithm, a deterministic unidirectional coding system that preserves anonymity while allowing linkage. Data were linked at the national level to allow the curation of duplicates and identification of all vaccines and infections, except for the Balearic Islands, Community of Madrid, and Navarre, which sent the data cross-matched and completely anonymized.

### Specification of the Target Trial

Our observational study emulated a hypothetical target trial to estimate the effect of the administration of at least 1 dose of MVA-BN vaccine for the prevention of MPXV infection. The target trial would start on 12 July 2022, and the eligible population would be men aged ≥18 years receiving HIV-PrEP with no prior MPXV infection or MVA-BN vaccination since the beginning of the mpox outbreak and regardless of having vaccination against smallpox during childhood.

In the target trial, eligible individuals would be randomly assigned to either the administration of a first dose of MVA-BN vaccine (regardless of the vaccine brand or the administration route) or to no administration of vaccine within strata defined by age and region. The outcome of interest would be laboratory-confirmed MPXV infection.

### Emulation of the Target Trial

We emulated the target trial with the linked observational data, starting on 12 July 2022 and ending on 12 December 2022 when the first region extracted the data for the study. We excluded individuals who started HIV-PrEP before it was included in the National Health System (November 2019), with missing date of infection, or with ≥3 doses of MVA-BN vaccine during the study period. Some regions did not have the information on sex within the HIV-PrEP registry, and it was assumed that all were males since nearly all HIV-PrEP users (99.7%) are men [23].

On each day between 12 July 2022 and 12 December 2022, we identified individuals who met the eligibility criteria and classified them as either having or not having received a first dose of MVA-BN vaccine that day. Each vaccinated person was matched to a randomly selected control among eligible individuals who had not received any dose of vaccine up to that date. Exact matching was performed, with replacement, on age (±5 years) and region. Vaccinated individuals could be matched as unvaccinated controls in the period up to 1 day before administration of the first dose.

The outcome of the study was laboratory-confirmed MPXV infection, with the date of the event defined as the earliest between the date of symptom onset or laboratory confirmation. For each matched pair, follow-up started on the day of administration of the first dose of MVA-BN vaccine and ended at the earliest of the date of event, death, or 12 December 2022. We followed a per-protocol approach to estimate VE; hence, we censored both members of a matched pair when the control was vaccinated.

We performed a secondary analysis restricting to pairs in which both members were younger than 50 years as a proxy of VE with no vaccination against smallpox during childhood, since individuals under 50 years should not have had the opportunity to get smallpox vaccines. We were unable to assess the VE of 2 MVA-BN doses because no infection was registered after the administration of a second dose. Likewise, VE of only 1 dose was equivalent to VE of at least 1 dose (the main analysis).

### Statistical Analyses

We computed the cumulative incidence (risk) curves of MPXV infection using the Kaplan–Meier estimator [24]. We computed the risk ratio (RR) overall and at different points in time: for days 0–6 or ≥7 days after administration of the first dose or, alternatively, for days 0–13 or ≥14 days. To compute risk and RRs ≥7 and ≥14 days after vaccination, we only used matched pairs in which both individuals were still at risk at 7 days (and 14) after time zero. We computed percentile-based 95% confidence intervals (CIs) using nonparametric bootstrapping with 500 samples [25]. We estimated VE as VE = (1 − RR) × 100. Analyses were performed with R software version 4.1.2 (R Foundation for Statistical Computing).

To test the impact of the analytical approach [26], we conducted a sensitivity analysis using the entire eligible population with time-varying vaccination status. We computed the number of events and time at risk by vaccination status, calendar week, age group, and region and estimated the adjusted incidence rate ratio (IRR) with Poisson regression. The detailed methodology and results of this analysis are found in the Supplementary Material.

The Institute of Health Carlos III Research Ethics Committee and the Community of Madrid Research with Drugs Ethics Committee approved the study.

## RESULTS

### Description of Study Participants

**Figure 1.**
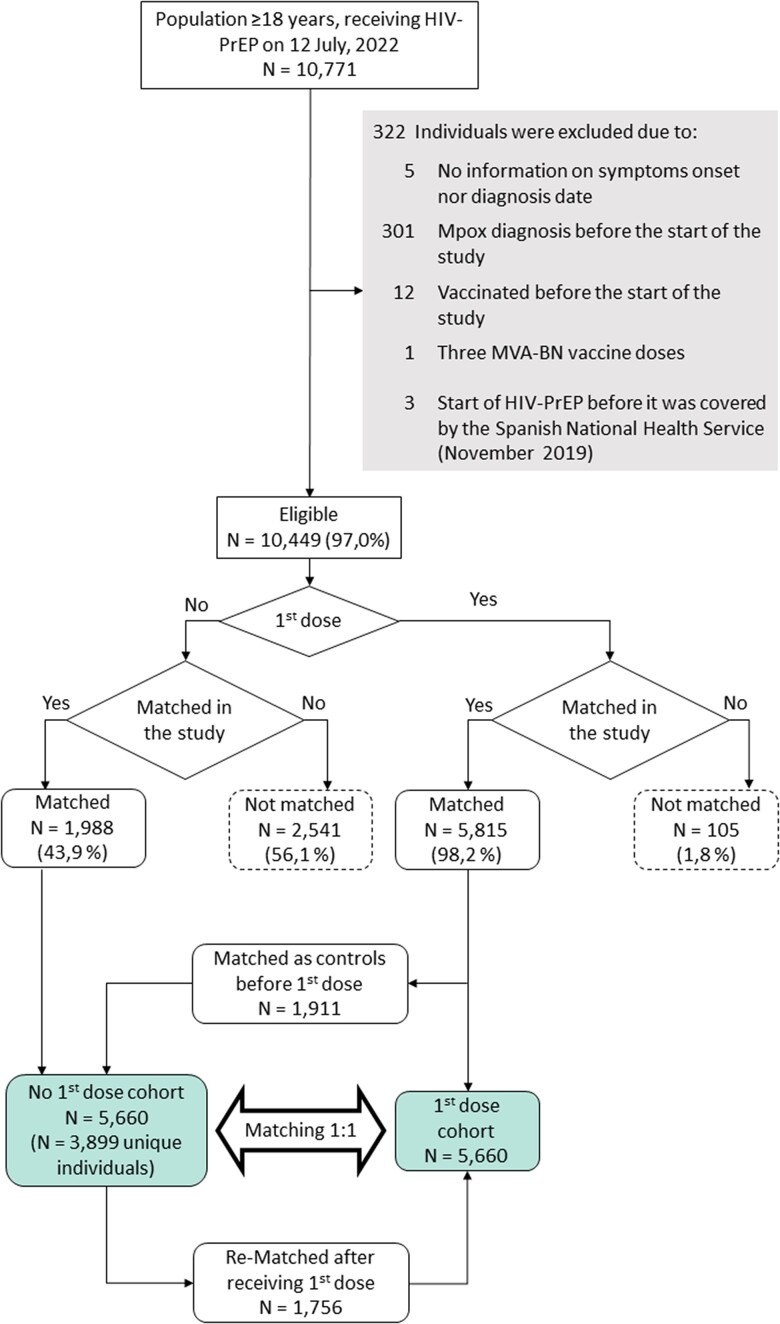
Sample selection flowchart. Abbreviations: HIV-PrEP, HIV pre-exposure prophylaxis; MVA-BN, modified vaccinia Ankara-Bavaria Nordic.

We identified 10 449 eligible individuals, of whom 5920 (56.7%) received a first dose of MVA-BN vaccine and 2014 (19.3%) received 2 doses. Both the initial and eligible populations had similar characteristics (Supplementary Table 1). We matched 5660 (95.6%) individuals who received a first dose of MVA-BN vaccine to the same number of controls who had not received vaccination against mpox up to the day when the vaccinated individual recieved that dose (Figure 1). The unvaccinated group included 3899 unique individuals, with a maximum number of repetitions of a matched control of 7. Censoring because the control received the first dose of MVA-BN vaccine occurred in 42.6% (n = 2412) of matched pairs.

Compared with the eligible population (Supplementary Table 1), individuals in the matched sample had similar age (median, 36 years; interquartile range [IQR], 31–43). Virtually no individuals received a dose of smallpox vaccine during childhood in the matched sample (n = 2, 0.0%) compared with the eligible population (n = 135, 1.3%). No hospitalizations, intensive care unit admissions, or deaths were recorded in the matched sample, while 19 and 11 mpox cases were hospitalized in the initial and eligible populations, respectively.

Table 1 shows the characteristics of the matched sample by vaccination status. The number of MPXV infections recorded was 43, with a higher number of cases among the unvaccinated (25 vs 18 in the vaccinated).

**Table 1.** Characteristics of Individuals in the Matched Sample by Vaccination Status

Characteristic	Vaccinated (n = 5660)		Unvaccinated (n = 5660)	
	n	%	n	%
Age group, y				
18–29	964	17.0	1003	17.7
30–39	2622	46.4	2607	46.1
40–49	1557	27.5	1534	27.1
≥50	517	9.1	516	9.1
Childhood smallpox vaccination				
Yes	1	0.0	1	0.0
No	44	0.8	48	0.8
Unknown	5615	99.2	5611	99.2
Autonomous region				
Andalusia	926	16.4	926	16.4
Asturias	37	0.7	37	0.7
Balearic Islands	361	6.4	361	6.4
Canary Islands	212	3.7	212	3.7
Castile and León	59	1.0	59	1.0
Castilla-La Mancha	59	1.0	59	1.0
Catalonia	2121	37.5	2121	37.5
Valencian Community	351	6.2	351	6.2
Extremadura	27	0.5	27	0.5
Galicia	252	4.5	252	4.5
Community of Madrid	830	14.7	830	14.7
Region of Murcia	129	2.3	129	2.3
Navarre	44	0.8	44	0.8
Basque Country	293	5.2	293	5.2
La Rioja	2	0.0	2	0.0
Mpox virus infection				
Yes	18	0.3	25	0.4
No	5642	99.7	5635	99.6
Mpox symptoms^a^				
Yes	18	100.0	25	100.0
No	0	0.0	0	0.0
Hospitalization^a^				
Yes	0	0.0	0	0.0
No	18	100.0	25	100.0
Admitted to intensive care unit^a^				
Yes	0	0.0	0	0.0
No	18	100.0	25	100.0
Death^a^				
Yes	0	0.0	0	0.0
No	18	100.0	25	100.0
MVA-BN product				
IMVANEX	340	6.0	-	-
JYNNEOS	3554	62.8	-	-
Unknown	1766	31.2	-	-
MVA-BN route of administration				
Intradermal (0.1 mL)	3502	61.9	-	-
Subcutaneous (0.5 mL)	1707	30.2	-	-
Unknown	451	7.9	-	-

Abbreviation: MVA-BN, modified vaccinia Ankara-Bavaria Nordic.

^a^Proportion is estimated with the total (100%) being the number of mpox virus infections.

**Table 2.** Number of Events, Estimated Risk, Risk Ratios, Vaccine Effectiveness, and 95% Confidence Intervals Overall and by Time Since Vaccination With 1 Dose of Modified Vaccinia Ankara-Bavaria Nordic Vaccine

Time Since Vaccination, days	Unvaccinated		Vaccinated		Risk Ratio (95% CI)	Vaccine Effectiveness (95% CI)
	Events	Risk Per 1000	Events	Risk Per 1000		
Overall	25	5.58	18	3.46	0.62 (0.31 to 1.24)	37.9% (−24.4 to 69.1)
0–6	8	1.46	11	2.02	1.38 (0.54 to 4.33)	−38.3% (−332.7 to 46.4)
0–13	13	2.47	15	2.82	1.14 (0.52 to 3.00)	−14.1% (−199.7 to 47.9)
≥7	17	4.13	7	1.44	0.35 (0.12 to 0.77)	65.0% (22.9 to 88.0)
≥14	12	3.12	3	0.65	0.21 (0.00 to 0.67)	79.3% (33.3 to 100.0)

Abbreviation: CI, confidence interval.

**Figure 2.**
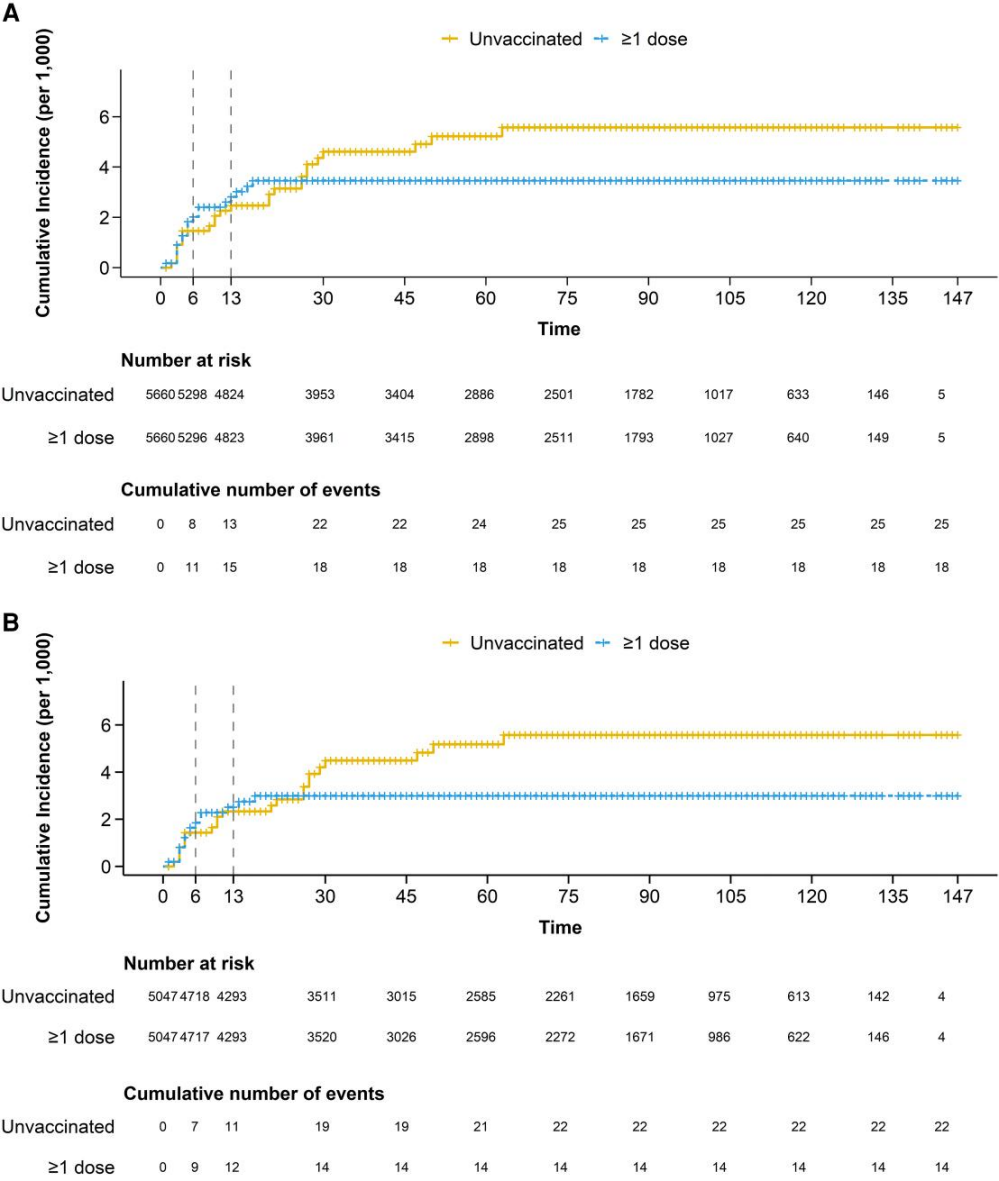
Estimated mpox virus infection risk in the sample of individuals vaccinated with at least 1 dose of Modified Vaccinia Ankara-Bavaria Nordic vaccine and matched unvaccinated controls overall (*A*) and in individuals aged <50 years (*B*).

Figure 2 *A* depicts the cumulative incidence in the vaccinated and unvaccinated groups. Median follow-up was 62 days (IQR, 24–97), with a maximum of 147 days. Cumulative incidence was 5.58 cases per 1000 individuals in the unvaccinated compared with 3.46 per 1000 in the vaccinated. Of 25 mpox cases among the unvaccinated, 8 (32%) and 13 (52%) cases were registered during the first 6 and 13 days, respectively. Of 18 mpox cases among the vaccinated, 11 (61%) and 15 (83%) were reported in the same period. The last mpox case was registered after 63 days of follow-up in the unvaccinated and after 17 days in the vaccinated.

### Effectiveness of 1 Dose of MVA-BN

During the study period, the overall estimated effectiveness of 1 dose of MVA-BN vaccine was 37.9% (95% CI, −24.4 to 69.1) (Table 2). During the first 6 and 13 days, the estimated VE was −38.3% (95% CI, −332.7 to 46.4) and −14.1% (95% CI, −199.7 to 47.9), respectively, showing a nonstatistically significant higher risk of MPXV infection in the vaccinated group. At ≥7 days post-vaccination, the estimated VE was 65.0% (95% CI, 22.9 to 88.0), and it increased to 79.3% (95% CI, 33.3 to 100.0) at ≥14 days.

### Results From Secondary and Sensitivity Analyses

We restricted the analysis to 5047 matched pairs in which both individuals were aged <50 years. Among vaccinated individuals, 14 mpox cases were registered compared with 22 cases among the unvaccinated. The last case of mpox was registered 63 days after enrollment in the unvaccinated and after 17 days in the vaccinated. The risk of MPXV infection was higher in unvaccinated individuals (5.6 per 1000) than in vaccinated individuals (3.0 per 1000; Figure 2*B*, Table 3). VE from 7 days post-vaccination onwards was 72.4% (95% CI, 20.9 to 94.4) and from 14 days onwards VE was 85.2% (95% CI, 41.1 to 100.0).

**Table 3.** Number of Events, Estimated Risk, Risk Ratios, Vaccine Effectiveness, and 95% Confidence Intervals Overall and by Time Since Vaccination With 1 Dose of Modified Vaccinia Ankara-Bavaria Nordic Vaccine Among Individuals Aged <50 Years

Time Since Vaccination, days	Unvaccinated		Vaccinated		Risk Ratio (95% CI)	VE (95% CI)
	Events	Risk Per 1000	Events	Risk Per 1000		
Overall	22	5.58	14	3.00	0.54 (0.25 to 1.03)	46.3 (−3.4 to 75.4)
0–6	7	1.44	9	1.86	1.29 (0.48 to 3.93)	−29.2 (−292.8 to 51.9)
0–13	11	2.34	12	2.52	1.08 (0.44 to 2.52)	−7.8 (−151.7 to 56.0)
≥7	15	4.15	5	1.15	0.28 (0.06 to 0.79)	72.4 (20.9 to 94.4)
≥14	11	3.26	2	0.48	0.15 (0.00 to 0.59)	85.2 (41.1 to 100.0)

Abbreviations: CI, confidence interval; VE, vaccine effectiveness.

The sensitivity analysis using the full eligible population and Poisson regression obtained similar results (Supplementary Table 2). Supplementary Figure 1 depicts the person-days of follow-up and the distribution of cases and incidence rates by vaccination status throughout the study period. Overall VE during the study period was 43% (95% CI, 7 to 65). No vaccine effect was observed during the first 6 and 13 days post-vaccination, respectively. From 7 days onwards, VE was 68% (95% CI, 35 to 84), and from 14 days onwards, the VE increased to 76% (95% CI, 39 to 90).

## DISCUSSION

Using a matched cohort study of a population receiving HIV-PrEP, we estimated that 1 dose of MVA-BN vaccine reduces the risk of MPXV infection by 65% from 7 days post-vaccination and by 79% from 14 days post-vaccination. Results were similar in a sensitivity analysis restricted to the population aged <50 years. These results confirm that MVA-BN vaccination is an effective prevention tool in a population at high risk of MPXV infection, at least shortly after vaccine administration, with the last detected MPXV infection at around 2 months of follow-up. Since the pre-exposure vaccination campaign in Spain began at a time when the incidence of mpox started to decrease, and has remained very low, the effectiveness at longer times since vaccination could not be assessed. The reduction in mpox incidence was most likely driven by an increase in the risk perception, the reduction in risky sexual practices during the outbreak, and the transmission dynamic of MPXV [27]; however, the high acceptability of MVA-BN vaccination and the effectiveness estimated in this study may have contributed to the suppression of transmission.

In our study, the risk of MPXV infection in the immediate days after vaccination was higher (though not statistically significant) in the vaccinated group. This could be explained by some misclassification of post-exposure vaccines as pre-exposure if people with a risk contact would seek vaccination even if they chose not to disclose such contact. Since most MPXV infection symptoms initiate within 5–7 days of exposure [28, 29], it is expected that estimates of VE after 7 days of vaccination are no longer affected by the inadvertent inclusion of post-exposure vaccinations. On the other hand, it could be that vaccination was sought preferentially by those most at risk of infection (since we could not obtain information on sexual behavior) or vaccination was erroneously perceived as granting protection, which could also result in an overall underestimation of the VE, as discussed later.

The majority of previous studies have reached similar or higher VE estimates. Two studies based on aggregated data conducted in the United States [16, 17] in males aged 18–49 years found a risk of MPXV infection more than 7 times higher (equivalent to 86% protection) in the unvaccinated compared with the vaccinated. One study in the United Kingdom [15] using the screening method [30] found a single-dose VE of 78%. Two studies in the United States using case-control designs have provided discordant estimates of VE with 1 dose of 72% [19] or 41% [18] among immunocompetent individuals, likely due to differences in the selection criteria for both cases and controls. Both studies also estimated VE of full vaccination with 2 doses of MVA-BN vaccine at 86% and 66%, respectively [18, 19].

Sagy et al conducted the only cohort study currently available in the literature to estimate the effectiveness of MVA-BN vaccination [20]. The study population was males who were HIV-PrEP users or were living with HIV and recently diagnosed with 1 or more STIs. The study estimated a VE (pre- and post-exposure) of 86%, which is probably an overestimate due to methodological limitations such as failure to identify equivalent time zero for both study groups, leading to important confounding by calendar time [21], and the exclusion of individuals vaccinated after 26 September 2022 (presumably also of their unvaccinated follow-up time), ignoring immortal time bias in observational studies [31]. To avoid misspecification of time zero, we emulated a target trial, as described in the literature [31, 32] and as widely used in the highest-quality studies on effectiveness of coronavirus disease 2019 vaccines [15, 33, 34].

The main strengths of our study are the careful specification of the start and end of the individual follow-up time and the dynamic matching to account for the time-changing baseline risk in the context of the mpox outbreak. Second, the availability of the vaccine indication allowed us to specifically estimate its effectiveness administered pre-exposure, although we cannot rule out certain misclassification, as discussed earlier, or data collection errors. Finally, the choice of individuals on HIV-PrEP as the study population has a higher chance of resulting in a group with homogenous sexual practices and behavior compared with studies that included diverse population groups. Moreover, because MVA-MB vaccine was actively recommended in this group (in some regions, even via short message service [SMS] advice), we ensure that all participants had the opportunity to be vaccinated, especially since it is a group with good demonstrated access to the healthcare system. This may have decreased the chance of confounding by preferential vaccine uptake in groups with different risks of exposure and the possible differential ascertainment of mpox infection.

Our study has some limitations. First, although we identified more than 10 000 eligible individuals with more than 700 MPXV diagnosed infections, a limited number of events were detected in the vaccinated group, which resulted in wide CIs. Moreover, we were not able to estimate the effectiveness of 2 doses of MVA-BN vaccine since all MPXV infections were registered in the first 2 months of follow-up when no second doses had been administered. The lack of hospitalizations or deaths prevented any estimate of effectiveness against these outcomes.

Second, we lacked any information on risk practices and behaviors. It is possible that individuals who sought MVA-BN vaccination were those at higher risk of mpox, for example, if individuals receiving HIV-PrEP due to living in a serodiscordant monogamous relationship or with a lower number of sexual partners decided not to vaccinate due to their lower risk. Also, it is possible that sexual behaviors changed following vaccination due to the perceived sense of protection. Both situations would result in an underestimation of the VE. In contrast, those more preoccupied with preventive measures in general could have higher acceptance of vaccination, which would overestimate VE.

Third, we did not have good-quality information about smallpox vaccine doses administered during childhood. However, the VE for those aged <50 years, for whom childhood smallpox vaccination was rare [35], yielded results that were similar to those from the main analysis, suggesting that the overall VE estimates are not significantly biased.

Finally, regarding the generalizability, our study was restricted to men receiving HIV-PrEP, and our estimates may not be valid for the general population or for immunocompromised individuals.

## CONCLUSIONS

The administration of 1 dose of MVA-BN vaccine pre-exposure reduces the risk of MPXV infection in individuals on HIV-PrEP, at least shortly after vaccination. The results indicate that vaccination is an important tool for prevention and control of mpox during an outbreak. However, more studies are needed to evaluate the protection conferred by 2 doses of MVA-BN vaccines and the duration of the protection.

## Supplementary Data

Supplementary materials are available at *Clinical Infectious Diseases* online. Consisting of data provided by the authors to benefit the reader, the posted materials are not copyedited and are the sole responsibility of the authors, so questions or comments should be addressed to the corresponding author.

## Supplementary Material

ciad645_Supplementary_Data

## Notes

***Author Contributions.*** S. M., A. D., A. L., V. H., and C. O. conceived the idea and elaborated the study protocol. A. D., V. H., C. O., T. V., and S. M. coordinated data collection. Authors from the public health administrations in the autonomous regions were in charge of implementing the protocol for data extraction and sharing. M. F., T. V., and S. M. were responsible for data curation. M. F. performed the statistical analysis and wrote the first draft of the manuscript under the supervision of S. M. and with support from A. D. and V. H. The guarantor is M. F. All authors were involved in interpretation of study results and critically reviewed the manuscript content.

***Acknowledgments.*** The authors thank all of the workers in the epidemiological departments, vaccination programs, laboratory centers, and the National Health Service in every region who made all data available and the vaccination campaign possible, alongside the work of the civil society organizations that helped to reach the individuals for whom the vaccine was recommended.

***Members of the Spanish MPOX Vaccine Effectiveness Study Group:*** José Antonio Lluch Rodrigo, Juan Juaneda, Rosa Carbo Malonda, Jacobo Mendioroz, Joan Colom Farran, Fernando González Carril, Lorea Oscoz Echevarría, María Teresa Otero Barros, Nuria Suárez Gaiche, José Angélica Gómez Martínez, Borja Alcázar Bastante, Rocío Sánchez Santos, Lucía Fuster Sanjurjo, María del Carmen Pacheco Martínez, Nancy Coromoto Cruz, Laura García Hernández, Óscar Guillermo Pérez Martín, Marcos Alonso García, María Ángeles Gutiérrez Rodríguez, Beatriz López Centeno, Mario Margolles Martins, Eva Martínez Ochoa, José Luis Jiménez-Murillo, Miguel Mingo Gómez de Celis, Inma Jarrín, Bernardo Guzmán, and Berta Suárez.
