# Supplementary material for: Effectiveness of Modified Vaccinia Ankara-Bavaria Nordic Vaccination in a Population at High Risk of Mpox: A Spanish Cohort Study
Source: Clin Infect Dis. 2023 Oct 21;78(2):476–83. doi: 10.1093/cid/ciad645 (PMC10874271; doi:10.1093/cid/ciad645)

# Effectiveness of Modified Vaccinia Ankara-Bavaria Nordic Vaccination in a Population at High Risk of Mpox: A Spanish Cohort Study

# Mario Fontán-Vela, Victoria Hernando, Carmen Olmedo, Ermengol Coma, Montse Martínez, David Moreno-Perez, Nicola Lorusso, María Vázquez Torres, José Francisco Barbas del Buey, Javier Roig-Sena, Eliseo Pastor, Antònia Galmés Truyols, Francisca Artigues Serra, Rosa María Sancho Martínez, Pello Latasa Zamalloa, Olaia Pérez Martínez, Ana Vázquez Estepa, Amós José García Rojas, Ana Isabel Barreno Estévez, Alonso Sánchez-Migallón Naranjo, Jaime Jesús Pérez Martín, Pilar Peces Jiménez, Raquel Morales Romero, Jesús Castilla, Manuel García Cenoz, Marta Huerta Huerta, An Lieve Dirk Boone, María José Macías Ortiz, Virginia Álvarez Río, María Jesús Rodríguez Recio, María Merino Díaz, Belén Berradre Sáenz, María Teresa Villegas-Moreno, Aurora Limia, Asuncion Diaz, and Susana Monge; the Spanish MPOX Vaccine Effectiveness Study Group*

**SUPPLEMENTARY MATERIAL**

Table of contents

[Description of the initial, eligible and matched populations 3](#_Toc153532722)

[**Table S1**. Baseline characteristics of the initial, the eligible and the matched sample 3](#_Toc153532723)

[Sensitivity analysis 6](#_Toc153532724)

[**Table S2**. Number of events, person-days of follow-up, estimated risk, adjusted incidence rate ratio (IRR), vaccine effectiveness (VE) and 95% confidence intervals (95%CI), of one dose of MVA-BN vaccine using adjusted Poisson regression models 7](#_Toc153532725)

[**Figure S1.** Time of follow-up (a), and number of events and incidence rate (b) by vaccination status and epidemiological week in the eligible population (N=10,449) 8](#_Toc153532727)

| Description of the initial, eligible and matched populations**Table S1**. Baseline characteristics of the initial, the eligible and the matched sample | | | | | | |
| --- | --- | --- | --- | --- | --- | --- |
|  | Initial sample (N=10,771) | | Eligible sample (N=10,449) | | Matched sample (N=11,320) | |
|  | n | % | n | % | n | % |
| Age, years |  | |  | |  | |
| 18-29 | 2,077 | 19.3 | 2,013 | 19.3 | 1,967 | 17.4 |
| 30-39 | 4,605 | 42.8 | 4,467 | 42.7 | 5,229 | 46.2 |
| 40-49 | 2,848 | 26.4 | 2,756 | 26.4 | 3,091 | 27.3 |
| ≥ 50 | 1,241 | 11.5 | 1,213 | 11.6 | 1,033 | 9.1 |
| Childhood smallpox vaccination |  | |  | |  | |
| Yes | 163 | 1.5 | 135 | 1.3 | 2 | 0.0 |
| No | 33 | 0.3 | 27 | 0.3 | 92 | 0.8 |
| Unknown | 10,574 | 98.2 | 10,287 | 98.4 | 11,226 | 99.2 |
| Autonomous Region |  | |  | |  | |
| Andalusia | 1,923 | 17.8 | 1,862 | 17.8 | 1,852 | 16.4 |
| Asturias | 53 | 0.5 | 52 | 0.5 | 74 | 0.6 |
| Balearic Islands | 768 | 7.1 | 686 | 6.6 | 722 | 6.4 |
| Canary Islands | 333 | 3.1 | 325 | 3.1 | 424 | 3.7 |
| Castile and León | 103 | 1.0 | 101 | 1.0 | 32 | 0.3 |
| Castilla-La Mancha | 76 | 0.7 | 76 | 0.7 | 118 | 1.0 |
| Catalonia | 2,925 | 27.3 | 2,813 | 26.9 | 4,242 | 37.5 |
| Valencian Community | 821 | 7.6 | 812 | 7.8 | 702 | 6.2 |
| Extremadura | 87 | 0.8 | 86 | 0.8 | 54 | 0.5 |
| Galicia | 412 | 3.8 | 407 | 3.9 | 504 | 4.4 |
| Community of Madrid | 2,493 | 23.1 | 2,473 | 23.7 | 1,660 | 14.7 |
| Region of Murcia | 194 | 1.8 | 193 | 1.8 | 258 | 2.3 |
| Navarre | 57 | 0.5 | 56 | 0.5 | 88 | 0.8 |
| Basque Country | 516 | 4.8 | 498 | 4.8 | 586 | 5.2 |
| La Rioja | 10 | 0.1 | 9 | 0.1 | 4 | 0.0 |
| MVA-BN vaccination |  | |  | |  | |
| Yes | 5,862 | 55.4 | 5,831 | 55.8 | 5,660 | 50.0 |
| No | 4,909 | 45.6 | 4,618 | 44.2 | 5,660 | 50.0 |
| MVA-BN vaccine product* |  |  |  |  |  |  |
| IMVANEX | 449 | 7.8 | 448 | 7.7 | 340 | 6.0 |
| JYNNEOS | 3,708 | 63.2 | 3,700 | 63.4 | 3,554 | 62.8 |
| Unkown | 1,705 | 29.0 | 1,683 | 28.9 | 1,766 | 31.2 |
| MVA-BN route of administration* |  |  |  |  |  |  |
| Intradermal (0.1 ml) | 3,756 | 64.1 | 3,745 | 65.2 | 3502 | 61.9 |
| Subcutaneous (0.5 ml) | 1,727 | 29.5 | 1,720 | 29.5 | 1,707 | 30.2 |
| Unknown | 379 | 6.4 | 366 | 6.3 | 451 | 7.9 |
| MPXV infection |  | |  | |  | |
| Yes | 738 | 6.8 | 431 | 4.1 | 43 | 0.4 |
| No | 10,033 | 93.2 | 10,018 | 95.9 | 11,277 | 99.6 |
| Mpox symptoms** |  | |  | |  | |
| Yes | 723 | 98.0 | 425 | 98.6 | 43 | 100.0 |
| No | 15 | 2.0 | 6 | 1.4 | 0 | 0.0 |
| Hospitalization** |  | |  | |  | |
| Yes | 19 | 2.6 | 11 | 2.5 | 0 | 0.0 |
| No | 719 | 97.4 | 420 | 97.5 | 43 | 100.0 |
| Admitted to ICU** |  | |  | |  | |
| Yes | 0 | 0.0 | 0 | 0.0 | 0 | 0.0 |
| No | 738 | 100.0 | 431 | 100.0 | 43 | 100.0 |
| Death** |  | |  | |  | |
| Yes | 0 | 0.0 | 0 | 0.0 | 0 | 0.0 |
| No | 738 | 100.0 | 431 | 100.0 | 43 | 100.0 |
| * Proportion is over the total number with Mpox vaccination  **Proportion is over the total number of MPXV infections | | | | | | |

# Sensitivity analysis

**Methods and results of the sensitivity analysis using Poisson regression**

To test the impact of the analytical approach, we alternatively addressed the study question using incidence rates and Poisson regression to estimate incidence rate ratios (IRR) and derive vaccine effectiveness (VE = (1-IRR)*100).

The exposure was defined as receiving at least one dose of MVA-BN vaccine, and vaccination status was assigned dynamically. All individuals started their follow-up into the study on 12 July 2022 as unvaccinated, and continued at risk in that status up to the earliest of: date of MPXV infection, date of death, date of administration of a first MVA-BN vaccine dose (regardless of indication as pre or post-exposure prophylaxis) or 12 December 2022. If a first MVA-BN vaccine dose was administered as pre-exposure prophylaxis before MPXV infection, death or 12 December 2022, the individual was classified as vaccinated and started contributing time at risk in that group up to the earliest of: date of MPXV infection, date of death, or 12 December 2022. Follow-up ended with an event when the censoring reason was MPXV infection. To assess vaccine effectiveness (VE) by number of days since dose administration, time since vaccination was further split into days 0 to 6 or ≥7 post-vaccination or, alternatively, into days 0 to 13 or ≥14 post-vaccination.

We aggregated the number of person-days of follow-up and the number of events in each vaccination status category by region (n=15), epidemiological week and age-group (18-29 / 30-39 / 40-49 / ≥50)

We used Poisson regression models to estimate the incidence rate ratio (IRR) with 95% confidence interval (95%CI), using the number of events as dependent variable, vaccination status as independent variable, and the logarithm of the number of person-days as offset. We estimated the crude IRR and the IRR adjusted by epidemiological week (using restricted cubic splines with three knots), age-group and region (as categorical variables).

| **Table S2**. Number of events, person-days of follow-up, estimated risk, adjusted incidence rate ratio (IRR), vaccine effectiveness (VE) and 95% confidence intervals (95%CI), of one dose of MVA-BN vaccine using adjusted Poisson regression models | | | | | | |
| --- | --- | --- | --- | --- | --- | --- |
| Vaccination status | | Events | Person-days | Cumulative incidence (per 10,000) | Adjusted* IRR (IC95%) | VE (95%CI) |
| Unvaccinated | | 411 | 978,730 | 4.20 | Ref. | Ref. |
| Vaccinated (time since vaccination) | Overall | 20 | 552,399 | 0.36 | 0.57 (0.35 ; 0.93) | 43% (7 ; 65) |
|  | 0-6 days | 11 | 34,013 | 3.23 | 1.30 (0.71 ; 2.38) | -30% (-138 ; 29) |
|  | 0-13 days | 15 | 73,464 | 2.04 | 0.94 (0.55 ; 1.59) | 6% (-59 ; 45) |
|  | ≥ 7 days | 9 | 514,117 | 0.18 | 0.32 (0.16 ; 0.65) | 68% (35 ; 84) |
|  | ≥ 14 days | 5 | 474,666 | 0.11 | 0.24 (0.10 ; 0.61) | 76% (39 ; 90) |
| *Adjusted by calendar week, age and region within Spain. | | | | | | |

## **Figure S1.** Time of follow-up (a), and number of events and incidence rate (b) by vaccination status and epidemiological week in the eligible population (N=10,449)

(a)


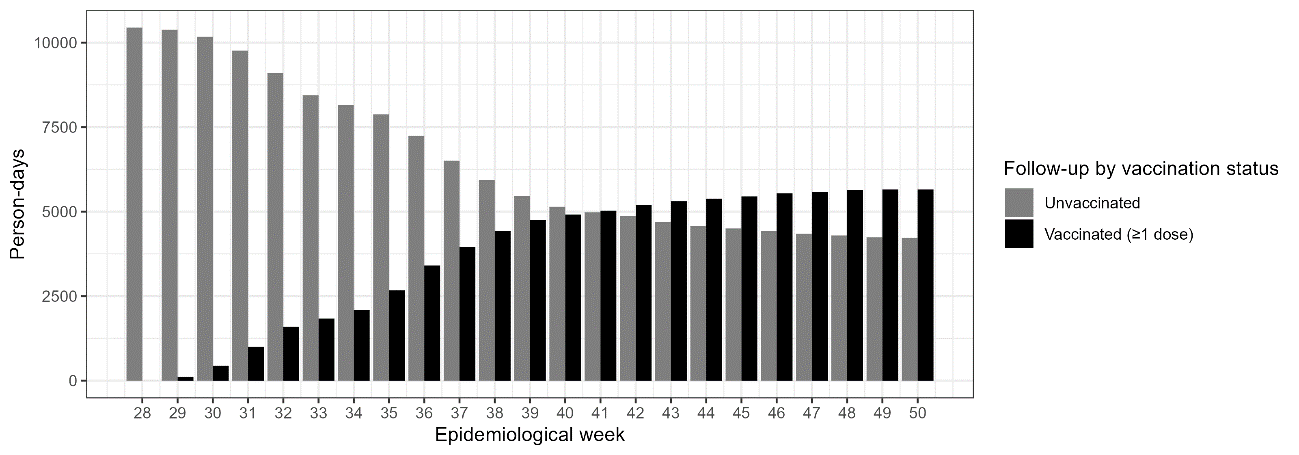


(b)


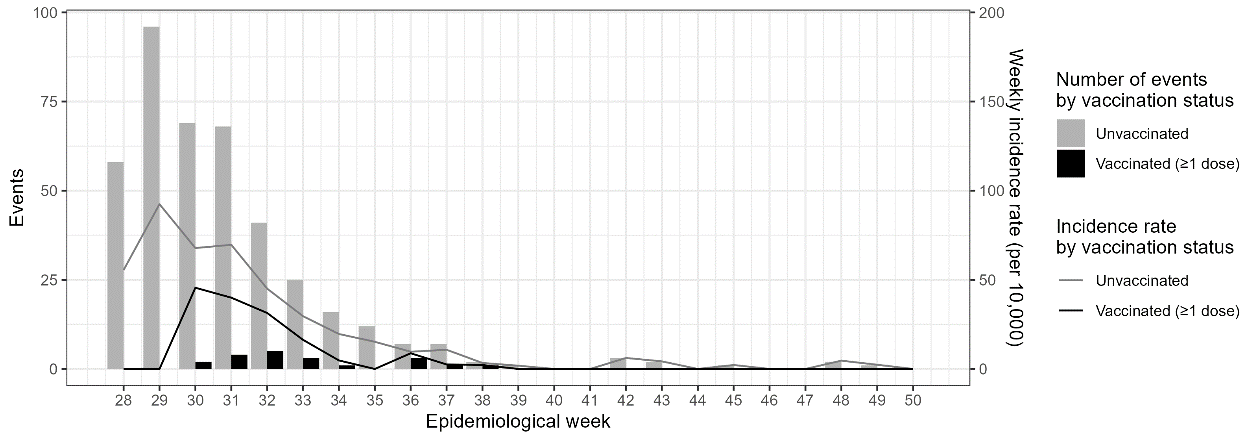

Supplement: ciad645_Supplementary_Data [file ciad645_supplementary_data.docx]
